# Incidence of medically attended influenza among residents of Shai-Osudoku and Ningo-Prampram Districts, Ghana, May 2013 – April 2015

**DOI:** 10.1186/s12879-016-2078-x

**Published:** 2016-12-13

**Authors:** Michael Preko Ntiri, Jazmin Duque, Meredith L. McMorrow, Joseph Asamoah Frimpong, Prince Parbie, Edem Badji, Ndahwouh Talla Nzussouo, Eve-Marie Benson, Michael Adjabeng, Erica Dueger, Marc-Alain Widdowson, Fatimah S. Dawood, Kwadwo Koram, William Ampofo

**Affiliations:** 1 Noguchi Memorial Institute for Medical Research, University of Ghana, Accra, Ghana; 2 Battelle Atlanta, Atlanta, Georgia USA; 3 Influenza Division, National Center for Immunization and Respiratory Diseases, U.S. Centers for Disease Control and Prevention, 1600 Clifton Rd NE, MS-A32, Atlanta, GA 30329 USA; 4 U.S. Public Health Service, Rockville, Maryland USA; 5 CTS Global Inc, El Segundo, California USA; 6 Ghana Health Service, Accra, Ghana

**Keywords:** Influenza, Respiratory, Burden, Rate, Children, Ghana, West Africa, Africa

## Abstract

**Background:**

Influenza vaccination is recommended by the World Health Organization for high risk groups, yet few data exist on influenza disease burden in West Africa.

**Methods:**

We estimated medically attended influenza-associated illness rates among residents of Shai-Osudoku and Ningo Pram-Pram Districts (SONPD), Ghana. From May 2013 to April 2015, we conducted prospective surveillance for severe acute respiratory illness (SARI) and influenza-like illness (ILI) in 17 health facilities. In 2015, we conducted a retrospective assessment at an additional 18 health facilities to capture all SONPD SARI and ILI patients during the study period. We applied positivity rates to those not tested to estimate total influenza cases.

**Results:**

Of 612 SARI patients tested, 58 (9%) were positive for influenza. The estimated incidence of influenza-associated SARI was 30 per 100,000 persons (95% CI: 13-84). Children aged 0 to 4 years had the highest influenza-associated SARI incidence (135 per 100,000 persons, 95% CI: 120-152) and adults aged 25 to 44 years had the lowest (3 per 100,000 persons, 95% CI: 1-7) (p < 0.01). Of 2,322 ILI patients tested, 407 (18%) were positive for influenza. The estimated incidence of influenza-associated ILI was 844 per 100,000 persons (95% CI: 501-1,099). The highest incidence of influenza-associated ILI was also among children aged 0 to 4 years (3,448 per 100,000 persons, 95% CI: 3,727 – 3,898). The predominant circulating subtype during May to December 2013 and January to April 2015 was influenza A(H3N2) virus, and during 2014 influenza B virus was the predominant circulating type.

**Conclusions:**

Influenza accounted for 9% and 18% of medically attended SARI and ILI, respectively. Rates were substantive among young children and suggest the potential value of exploring the benefits of influenza vaccination in Ghana, particularly in this age group.

**Electronic supplementary material:**

The online version of this article (doi:10.1186/s12879-016-2078-x) contains supplementary material, which is available to authorized users.

## Background

Influenza is an important contributor to acute respiratory infection (ARI) - a leading cause of morbidity, mortality and economic loss worldwide [1]. A review of seasonal influenza epidemiology in sub-Saharan Africa found that 10% (range: 1%-25%) of outpatient acute respiratory cases and 7% (range: 1%-16%) of children hospitalized with ARI tested positive for influenza [2]. The impact of seasonal and pandemic influenza could be substantial in Africa due to the prevalence of other infections and comorbidities that could increase the severity of influenza disease [3, 4]. During 2006 to 2010, influenza surveillance capacity increased substantially in sub-Saharan Africa [5]. There are now 24 World Health Organization (WHO) designated National Influenza Centers in Africa and 10 African countries regularly report influenza surveillance data to the Global Influenza Surveillance and Response System (GISRS) [6]. Despite these advances, there are few data describing influenza disease burden in West African countries.

During 2012 in Ghana, lower respiratory tract infections were the leading cause of death [7]. In 2013, Noguchi Memorial Institute for Medical Research (NMIMR) of the University of Ghana, Ghana Health Service and the U.S. Centers for Disease Control & Prevention (CDC) established health facility–based surveillance for influenza and other respiratory viruses among residents of Shai-Osudoku and Ningo-Prampram Districts (SONPD) in the Greater Accra Region. The NMIMR serves as Ghana’s National Influenza Centre (NIC). The Dodowa Health and Demographic Surveillance System (HDSS), established in 2005, monitors the demographics of 121,943 residents [8] of SONPD. Surveillance data indicate that influenza transmission is year-long with peaks during the rainy seasons although further surveillance to ascertain seasonality is needed. The current immunization program does not include the use of seasonal influenza vaccines in Ghana. Following the 2009 influenza A(H1N1) pandemic, it became clear that data on influenza were needed to guide public health policies and actions to lessen the impact of influenza on populations in West Africa. We present incidence estimates of medically attended influenza in a rural peri-urban area of Ghana through health facility-based prospective and retrospective surveillance.

## Methods

### Surveillance sites

#### SARI and ILI Surveillance

In 2012, a health utilization survey (HUS) identified the health facilities where SONPD residents frequently sought care and this information was used to identify the study surveillance sites [9]. Only residents of SONPD were included in the study regardless of whether the surveillance site was located in or outside the SONPD. Patients with an HDSS identification number and/or a SONPD address were identified as a resident. In early 2013, we established prospective severe acute respiratory illness (SARI) and influenza-like illness (ILI) surveillance in nine health facilities: three hospitals, three clinics and three community health centers. We conducted SARI surveillance in the three hospitals and ILI surveillance in all nine facilities, collecting both epidemiologic data and laboratory specimens from eligible case-patients. Seven of these nine facilities were located within SONPD and two were in adjacent districts (Lower Manya District and North Tongu District). Although the study period started in May 2013, prospective surveillance was established in March 2013. The two months between the start of surveillance and the start of the study period served to address operational mishaps and ensure data quality.

The 2012 HUS identified another eight community health centers in SONPD with very few (e.g., 1-10) patient visits per week. Due to their low patronage and remote location, we collected epidemiologic data from these eight ILI surveillance sites but did not collect laboratory specimens. Hence, there were a total 17 study surveillance sites: 9 collecting both epidemiologic data and laboratory specimens from eligible case-patients and 8 collecting epidemiologic data only from April 2013 to May 2015.

### Retrospective record review

**Fig. 1:**
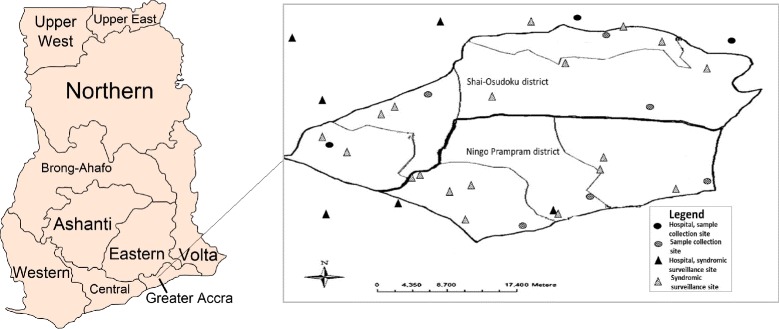
Map of Ghana and geographic distribution healthcare facilities in which virologic and/or syndromic surveillance were conducted to assess the burden of medically attended influenza among residents of Shai-Osudoku and Ningo-Prapram districts, May 2013- April 2015. Image attribution: By Thfc - Own work, CC BY-SA 3.0, https://commons.wikimedia.org/w/index.php?curid=20018233

In 2015, we conducted an assessment of the catchment area and decided to perform a retrospective record review of an additional 18 health facilities (14 inside and 4 outside SONPD) which had been part of the 2012 HUS to capture all SARI and ILI patients for this study [9]. We reviewed consulting room registers, patient folders and admission records for period May 2013 to April 2015 and captured all data electronically. Laboratory specimens from these SARI and ILI patients were not available for testing. Figure 1 depicts the geographic distribution of all of the healthcare facilities included in this study and differentiates between sites where specimens were collected and where only syndromic data were collected. In all, nine hospitals were included in the study. The 2012 HUS showed that >99% of SONPD residents sought care at one of these hospitals.

### Eligibility, consenting and recruitment

ILI was defined as a respiratory illness with history of fever or measured axillary temperature ≥37.5 °C and cough with onset within the last 10 days. The WHO recommended case definition for ILI does not include a history of fever [10]. SARI was defined as an ILI requiring hospitalization. Eligible subjects were patients aged ≥1 month, resident of SONPD, who sought care at a study site and met one of the above case definitions. Patients aged <1 month were excluded because investigators felt it was culturally inappropriate to ask caregivers for their participation in the study. Study staff were present in the health facilities Monday through Friday during business hours. Staff reviewed weekend and after hour log books to identify all SARI and ILI patients. Patients who resided outside SONPD were excluded from the study.

All eligible SARI and the first five eligible ILI patients per site who provided consent were enrolled weekly. ILI case enrolment began at the beginning of the week and ended as soon as five patients had been enrolled irrespective of day of the week. Study staff explained the risks and benefits of study participation to eligible participants prior to enrolment. Participants who agreed to be part of the study were asked to sign a written consent form. For participants aged 5-17, parent/legal guardian consent and participant assent were also obtained. For participants aged <5 years, only parent/legal guardian consent was obtained.

### Ethical considerations

The surveillance protocol was reviewed and approved by the scientific and technical committee and the institutional review board of NMIMR (054/12-13). CDC granted a non-research determination (NRD#2013 6261).

### Data and specimen collection

Screening log books were used to record total attendance as well as total number of ILI and SARI patients at all 17 sites. Trained field staff, which included physicians, nurses, midwives and research assistants, used a structured questionnaire to capture clinical and demographic information from enrolled participants using a personal digital assistant (PDA) (Additional file 1: Figure SA). The age, gender, weight, date of illness onset, and date of visit were recorded for all SARI and ILI patients identified. In addition, date of admission and duration of hospitalization were recorded for SARI patients. Weight-for-age was calculated and categorized according to the WHO Child Growth Standards [11]. Data from PDAs were transferred electronically to a server at NMIMR on a biweekly basis. Trained healthcare workers collected nasopharyngeal and/or oropharyngeal swabs from enrolled patients and placed them in a single vial of transport medium (Becton Dickinson and Company, Franklin Lakes, New Jersey, USA). Specimens were transported within 24 hours in a cool box with ice packs to the NIC. Depending on the time of day the specimens arrived, they were either tested right away or frozen to be tested later. Specimens that were not sent to the NIC within 24 hours were stored on-site in liquid nitrogen tanks and transferred to the NIC in cool boxes.

### Virologic testing

Viral ribonucleic acid (RNA) was extracted using the QIAamp® Viral RNA Mini Kit (Qiagen, Hilden, Germany) according to manufacturer’s recommendations. Influenza virus was detected using standardized real-time reverse-transcription polymerase chain reaction (rRT-PCR) protocols from CDC (13). The rRT-PCR assays were performed with AgPath One-Step rRT-PCR kit (Thermo Fisher Scientific, Inc., Waltham, Massachusetts, USA) on the Applied Bios systems 7500 fast rRT-PCR instrument (Thermo Fisher Scientific, Inc., Waltham, Massachusetts, USA).

### Data analyses

Annual incidence rates were calculated using population denominators obtained from the HDSS [8]. Residents are enumerated in the HDSS annually and we used the population for year 2014. We estimated the number of influenza cases among those not tested by multiplying the percent positive among those tested by the number not tested; we did this first by using age-group specific positivity rates and second by age-group and month-specific positivity rates. The age groups used were 0 to 4, 5 to 14, 15 to 24, 25 to 44, 45 to 64 and >65 years. This same age grouping is used in the HDSS. We estimated rates of influenza-associated SARI and ILI by adding the numbers testing positive to those estimated to be positive among non-tested and dividing by the population. We calculated the 95% confidence intervals (lower and upper limits) for the proportion of patients tested with influenza-positive specimens and applied these to those who were not tested. The group referred to as “not tested” from this point forward includes all SONPD residents who were identified as SARI or ILI patients during the study period but did not have a laboratory specimen for influenza testing either because they were identified through the retrospective record review or during the prospective routine surveillance but were not tested for other reasons. The age group 0-4 years was not adjusted for the exclusion of infants <1 month because there were no population estimates for infants aged <1 month.

We calculated medians, interquartile ranges and rates with associated 95% confidence intervals using Microsoft Excel© (Microsoft Corporation, Redmond, WA). We calculated crude odds ratios and used the Wilcoxon Rank Sum test to compare medians in SAS version 9.3 (SAS Institute, Cary, NC) and used Fisher and mid-p exact tests to compare rates in OpenEpi (Dean AG, Sullivan KM, Soe MM., Emory University, Atlanta, GA). Results were considered statistically significant if the associated two-sided p-value was <0.05.

## Results

### Study population

**Fig. 2:**
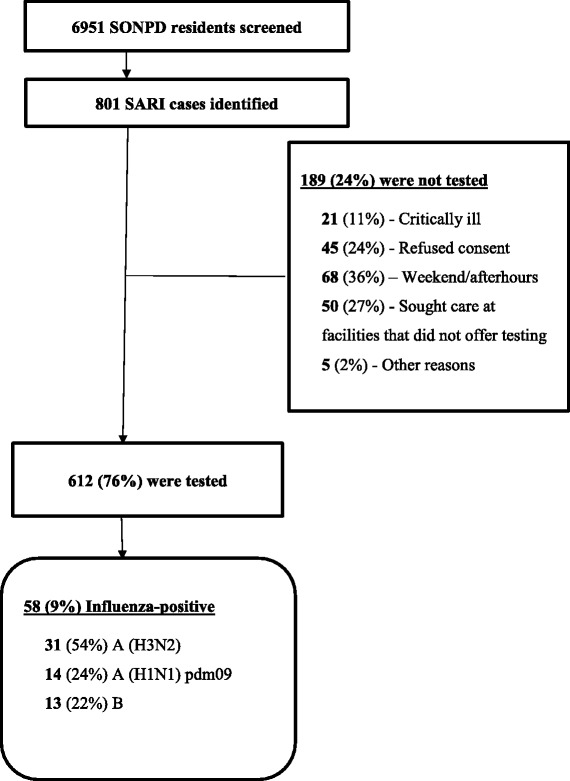
Total severe acute respiratory illness (SARI) patients identified and tested in Shai-Osudoku and Ningo-Prampram Districts (SONDP), May 2013 – April 2015

Between May 1, 2013 through April 30, 2015, there were 801 SARI patients among SONPD residents, 612 (76%) of which were tested for influenza. Approximately half (292/612) had a history of fever but no recorded temperature documenting a fever. The median age of SARI patients tested was 3 years (interquartile range [IQR]: 1-9 years) while that of SARI patients not tested was 9 years (IQR: 2-30 years) (*p* < 0.01) (Table 1). Patients were not tested because they were admitted and/or discharged on weekends or afterhours (*n* = 68, 36%), sought care at a facility that did not offer testing (*n* = 50, 27%) refused consent (*n* = 45, 24%), or were critically ill (*n* = 21, 11%) (Fig. 2). The median duration of hospitalization among SARI patients tested was 7 days (IQR: 3-9 days) and among SARI patients who were not tested was 1 day (IQR: 1-2 days) (Table 1).

**Table 1:** Characteristics of influenza-like illness (ILI) and severe acute respiratory illness (SARI) patients in Shai-Osudoku and Ningo-Prampram Districts, May 2013 – April 2015

	ILI			SARI		
	Tested (N = 2,322)	Not tested (N = 9,544)	Influenza-positive	Tested (*N* = 612)	Not tested (*N* = 189)	Influenza-positive
Age group, years (n, %)					<0.01	
0 to 4	1,355 (58)	5,161 (54)	203 (15)	386 (63)	74 (37)	32 (8)
5 to 14	433 (19)	2,026 (21)	108 (25)	108 (18)	28 (14)	14 (13)
15 to 24	163 (7)	677 (7)	36 (22)	27 (4)	26 (13)	4 (15)
25 to 44	231 (10)	979 (10)	41 (18)	49 (8)	31 (16)	1 (2)
45 to 64	100 (4)	440 (5)	15 (15)	30 (5)	21 (11)	5 (17)
≥65	40 (2)	261 (3)	4 (10)	12 (2)	19 (10)	2 (17)
Influenza-positive (n, %)	407 (18)			58 (9)		
Duration of symptoms prior to seeking health care-days^a^						
Median	3	3		2	0	
Interquartile Range	2-4	1-3		1-4	0-0	
Duration of hospitalization (SARI)- days^b^						
Median	NA	NA		7	1	
Interquartile Range	NA	NA		3-9	1-2	

^a^Missing data: ILI Tested = 51%, ILI Not tested = 23%, SARI Tested = 1 record missing 0%, SARI Not tested = 6%

^b^Missing data: SARI Tested = 5%, SARI Not tested = 20%

Of 11,866 eligible SONPD residents with ILI, 2,322 (20%) were tested. Half (52%) had a history of fever but no recorded temperature documenting a fever. The median age of ILI patients tested was 3 years (IQR: 1-12 years) while that of ILI patients not tested was 2 years (IQR: 0-10 years) (p <0.01). The median number of days between onset of symptoms and seeking medical care among ILI patients tested was 3 (IQR: 2-4 days) and among ILI patients who were not tested was 3 (IQR: 1-3) (Table 1).

### Virologic testing

Of 612 SARI patients tested, 58 (9%) were positive for influenza viruses. Among the influenza-positive cases, 31 (54%) were identified as influenza A(H3N2) virus, 14 (24%) as influenza A(H1N1) pdm09 virus, and 13 (22%) as influenza B virus. The median age of SARI patients testing positive for influenza was 4 years (IQR: 1-12 years) and of SARI patients testing negative for influenza was 3 years (IQR: 1-9 years) (*p* =0.02). Among hospitalized children aged 1 to 4 years, those who tested positive for influenza were more likely to be low weight-for-age than children who were influenza-negative (odds ratio: 3.3, 95% confidence interval [CI]: 1.3-10.3, *p* = 0.04).

**Fig. 3:**
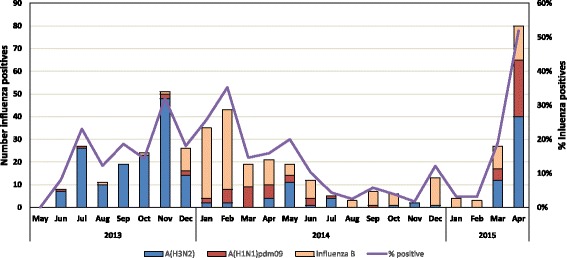
Distribution of influenza virus types and subtypes among influenza like illness (ILI) and severe acute respiratory illness (SARI) patients in Shai-Osudoku and Ningo-Prampram Districts, May 2013 - April 2015

Of 2,322 ILI patients tested, 407 (18%) were positive for influenza viruses; of these, 196 (48%) were influenza A(H3N2) virus, 53 (13%) influenza A(H1N1) pdm09 virus, and 158 (39%) influenza B virus. The median age of ILI patients testing positive for influenza was 5 years (IQR: 2-13 years) and of ILI patients testing negative for influenza was 3 years (IQR: 1-12 years) (*p* < 0.01). Although the predominant circulating subtype during May to December 2013 and January to April 2015 was influenza A(H3N2) virus, influenza B virus was the predominant circulating type during 2014. During 24 months of surveillance, there were influenza-positive specimens in 23 months (Fig. 3).

### Incidence of influenza-associated SARI and ILI

The incidence of influenza-associated SARI was 30 per 100,000 persons (95% CI: 13 to 84). The annual incidence was highest among children aged 0 to 4 years (135 per 100,000 persons, 95% CI: 120-152) and dropped to a low among adults aged 25 to 44 years (3 per 100,000 persons, 95% CI: 1-7) (*p* < 0.01) before rising slightly to 28 per 100,000 persons (95% CI: 21-36) among those >65 years of age. The rate of influenza-associated SARI among all ages during the first year of the study (28 per 100,000 persons, 95% CI: 10-87) was similar to the second year of the study (32 per 100,000 persons, 95% CI: 16-81) (*p* = 0.61) (Table 2).

**Table 2:** Estimated annual incidence of influenza-associated influenza-like illness (ILI) and severe acute respiratory illness (SARI) in Shai-Osudoku and Ningo-Prampram Districts, May 2013 – April 2015

	Incidence of influenza-associated ILI and SARI (95% CI)		
	Number of cases per 100,000 persons		Population denominator^b^
	ILI	SARI	
Overall (age^a^)	895 (854- 937)	33 (26 – 42)	121,943
Overall (age-month^┼^)	844 (501 – 1099)	30 (13 – 84)	
Age Group (age^a^)			
0 to 4 years	3,811 (3,727 – 3,898)	135 (120– 152)	12,807
5 to 14 years	1,026 (983 – 1,071)	30 (23 – 39)	29,888
15 to 24 years	356 (331 – 383)	16 (12 – 23)	26,049
25 to 44 years	327 (303 – 353)	3 (1 – 7)	32,865
45 to 64 years	285 (263 – 309)	33 (26 – 42)	14,205
≥65 years	246 (225 –268)	28 (21 – 36)	6,129
Year of Study (age^a^)			
Year 1 (May 2013 – April 2014)	1080 (707 - 1367)	28 (10 - 87)	
Year 2 (May 2014 – April 2015)	608 (296 – 831)	32 (16 - 81)	

Number positive among those not tested determined using age-group^a^ or age-group-month^┼^ specific positivity rates among those tested (see Methods)

^b^Population for year 2014 (Dodowa Health and Demographic Surveillance System)

The incidence of influenza-associated ILI was 844 per 100,000 persons (95% CI: 501-1,099). Children 0 to 4 years of age had the highest incidence of medically attended influenza-associated ILI (3,811 per 100,000 persons, 95% CI: 3,727-3,898). During the first year of the study, the rate of medically attended influenza-associated ILI was higher (1,080 per 100,000 persons, 95% CI: 707-1,367) than during the second year (608 per 100,000 persons, 95% CI: 296-831) (*p* < 0.01) (Table 2).

## Discussion

The incidence of influenza-associated hospitalizations and outpatient visits was highest among children aged 0 to 4 years in SONPD from May 2013 to April 2015. This is consistent with Nair et al.’s finding that the global burden of illness attributable to influenza in young children is substantial [12]. A study in South Africa found that children aged <1 year and adults aged >75 years had the highest rates of influenza-associated respiratory hospitalization estimated at 255 per 100,000 person-years and 380 per 100,000 person-years, respectively [13]. Similarly, Emukule et al. estimated the annual incidence of hospitalized influenza-associated SARI among children aged <5 years in Kenya to be between 180 and 390 cases per 100,000 person-years [14].

Influenza circulated year-round in the districts during the study period; this is consistent with studies summarizing influenza surveillance data from West Africa [5, 8]. During the 24 month study period, the primary circulating subtype in SONPD was influenza A(H3N2) virus. For this same time period, WHO’s GISRS reported influenza A(H3N2) virus as the predominant subtype circulating in West Africa based upon data received from Ghana and 5 other West African countries [6]. A summary of global circulation of influenza viruses using data from 85 countries found that tropical settings, like Ghana, have year-round influenza activity more often than temperate and subtropical sites [15]. While influenza-associated SARI rates were similar for both study years, the rates of medically attended influenza-associated ILI varied significantly by year. Although the reason for this variation is unknown, it could be that this is representative of the true burden of influenza illness or a reflection of year-to-year variability in health-care seeking behavior due to availability of public services or changes in the local economy.

Our study only assessed medically attended SARI and ILI. We have no doubt underestimated the true incidence of influenza-associated illness in this community because we did not measure non-medically attended ILI or SARI. Although we used the 2012 HUS to select the study sites and are confident to have captured close to all medically attended SARI, we have less certainty about having captured all medically attended ILI among SONPD residents. A study comparing hospitalized influenza-associated SARI rates to non-hospitalized influenza-associated SARI rates highlighted the importance of healthcare seeking behaviour when calculating influenza-associated disease burden estimates, particularly in low and middle-income countries [16]. Moreover, although the elderly are at greater risk for influenza-associated complications and hospitalizations [17], we found that persons >65 years had rates of influenza-associated SARI similar to other adults, though our numbers were very small. This outcome may be due to various healthcare access barriers faced by the elderly despite Ghana’s National Health Insurance Scheme which exempts them from paying annual premiums [18]. We also decided to exclude infants <1 month old in our study. This limits the findings for age group aged 0-5 years, likely underestimating the true burden of influenza illness among the very young. Although prospective surveillance was conducted at a number of surveillance sites, we relied on review of medical records and registers to assess SARI and ILI in sites where we did not conduct prospective surveillance. It is possible that incomplete recording of fever, cough or duration of symptoms could have reduced detection of patients meeting the surveillance case definition.

This study describes the burden of medically attended influenza-associated illness in a population with continuous demographic surveillance. In some instances, age-group and month specific positivity rates were unstable because of small numbers. Using a 2-sided significance level likely underestimated variability, leading to narrow confidence intervals. Our methods applied the proportion influenza-positive among those tested to those not tested in order to estimate the total number of influenza cases by age-group. ILI cases were systematically sampled to limit potential bias between those tested and not tested. There were, however, statistically significant differences in the median ages of tested versus non-tested groups among SARI and ILI patients. This is a study limitation because there is no way to know if this resulted in an over- or under-estimation of the true burden of disease. In addition, more data are needed to estimate the burden of influenza-associated illness among high-risk groups, including pregnant women, those aged 0-6 months and HIV-infected individuals. We are currently conducting separate studies in SONPD to address some of these data gaps.

## Conclusions

In this population in Ghana, influenza-associated ILI and SARI has the highest burden among children aged 0 to 4 years. More data are needed to improve influenza disease burden estimates, including estimating non-hospitalised severe influenza-associated illness by age, especially in the elderly and specific high-risk groups. Our findings suggest the value of modelling the number of cases and costs that could be averted through influenza vaccination of high-risk target groups identified by the WHO.

## Additional file

Additional file 1:
**Figure SA.** Enrolment process for severe acute respiratory illness (SARI) and influenza like illness (ILI) in Shai-Osudoku and Ningo-Prampram Districts, Ghana, 2013-2015. (DOCX 34 kb)

## Abbreviations

ARI: Acute respiratory infection
CDC: U.S. Centers for Disease Control and Prevention
CI: Confidence interval
GISRS: Global Influenza Surveillance and Response System
HDSS: Health and Demographic Surveillance System
HUS: Health utilization survey
ILI: Influenza-like illness
IQR: Interquartile range
NIC: National Influenza Centre
NMIMR: Noguchi Memorial Institute for Medical Research
NRD: Non-research determination
PDA: Personal digital assistant
RNA: Viral ribonucleic acid
rRT-PCR: Real-time reverse-transcription polymerase chain reaction
SARI: Severe acute respiratory illness
SONPD: Shai-Osudoku and Ningo Pram-Pram Districts
WHO: World Health Organization
