# Supplementary material for: Incidence of medically attended influenza among residents of Shai-Osudoku and Ningo-Prampram Districts, Ghana, May 2013 – April 2015
Source: BMC Infect Dis. 2016 Dec 13;16:757. doi: 10.1186/s12879-016-2078-x (PMC5155389; doi:10.1186/s12879-016-2078-x)
Supplement: Additional file 1: — Figure SA. Enrolment process for severe acute respiratory illness (SARI) and influenza like illness (ILI) in Shai-Osudoku and Ningo-Prampram Districts, Ghana, 2013-2015. (DOCX 34 kb) [file 12879_2016_2078_MOESM1_ESM.docx]

**IDENTIFICATION**

Using admission logbook, identify suspect SARI or ILI patient(s)

**SCREENING**

Confirm eligibility using screening logbook

*Eligible*

*Not Eligible*

**STOP!**

Criteria not met

**CONSENTING & ENROLLMENT**

Obtain consent & assent from patient/ legal guardian

For ILI only: first five eligible enrolled per week

*No Consent*

**STOP!**

Criteria not met

*Consent/Assent (assign study ID)*

**QUESTIONNAIRE**

Using PDA/Questionnaire, gather data from patient

**SAMPLE COLLECTION**

Obtain nasopharyngeal and/or oropharyngeal swabs and document in lab form & lab logbook

**OUTCOME INFORMATION**

Record case outcome on PDA/questionnaire and screening logbook
